# Supplementary material for: Systematic Review of Molecular Targeted Therapies for Adult-Type Diffuse Glioma: An Analysis of Clinical and Laboratory Studies
Source: Int J Mol Sci. 2023 Jun 21;24(13):10456. doi: 10.3390/ijms241310456 (PMC10341773; doi:10.3390/ijms241310456)
Supplement: Supplementary file 1 [file ijms-24-10456-s001.zip › ijms-2376896-supplementary.pdf]

Supplementary Table S1. Detailed Study Design of Clinical Studies Implementing Molecular Targeted Therapies in Glioma

Study Author	Year	Tumor Details	Molecular target	Intervention	Patient Age(s)*	N	Study Design	Quality Rating
**Protein Kinase Pathways**								
Berzero et al	2021	BRAF-V600 Mutant Gliomas	RAF + MEK	Vemurafenib, Dabrafenib, Cobimetinib, Trametinib	Median age 36	28	Retrospective Cohort	Level IV
Butowski et al	2010	Newly diagnosed GBM or GS with a biopsy or resection 5 weeks prior to treatment	Protein kinase C-beta + PI3K/AKT pathway	Enzastaurin + TMZ	> 18	12	Phase I Trial	Level IV
Chinnaiyan et al	2013	Newly diagnosed GBM	mTOR	Everolimus + TMZ + RT	Median age 57.6	35	Phase I/II Trial	Level IV
Drobysheva et al	2017	Pilocytic astrocytomas	BRAF + MAPK	Dabrafenib +/- Trametinib	15-Jan	5	Case Series	Level VI
Franceschi et al	2012	Histologically documented GBM and IDH-mutant Glioma	Src kinase	Dasatinib	Median age 54.8	26	Phase I/II Trial	Level IV
Fusco et al	2021	3/4 cases IDH-wt and BRAFV600E, 1/4 cases IDH1 mutant and BRAFV600E	BRAF + MEK	Dabrafenib + Trametinib	23.5 ± 6.40	4	Case Series	Level IV
Hottinger et al	2019	BRAF-mutated glioma	MAPK + ERK	Dabrafenib + Trametinib	Median ages 31 and 40	2	Case Report	Level VI
Johanns et al	2018	IDH-wt GBM	BRAF + MEK	Dabrafenib + Trametinib	28 + 24	2	Case Series	Level VI
Kaley et al	2018	GBM, anaplastic astrocytoma, pleomorphic xanthoastrocytoma, anaplastic ganglioma, pilocytic astrocytoma, HGG	BRAF	Vemurafenib	18-81	24	Phase II Trial	Level IV
Kanemaru et al	2019	GBM with BRAF V600E, TERT, and CDKN2A mutations	BRAF + MEK	Dabrafenib + Trametinib	Median age 57	1	Case Report	Level VI
Kebir et al	201	5 IDH-wt GBM;	Multitarget	Regorafenib	18–69	6	Retrospectiv	Level

	9	1 IDH-mutant	kinase				e Cohort	IV
Kleinschmidt-DeMasters et al	2015	19 IDH-wt GBM; 1 IDH-mutant Astrocytoma	BRAF V600E kinase	Vemurafenib	10–82	20	Retrospective Cohort	Level IV
Lapointe et al	2020	14 IDH-wt; 1 IDH-mutant	mTORC1/2	Vistusertib	Median age 66	15	Phase I Trial	Level IV
Lee et al	2012	Histology confirmed GBM	Multitarget kinase + mTOR	Sorafenib + Temsirolimus	Median age 50	31	Phase I/II Trial	Level IV
Lombardi et al	2019	Histology confirmed GBM	Multitarget kinase + mTOR	Regorafenib	54.8–58.9	119	Phase II Trial	Level II
Mason et al	2012	Newly diagnosed, histologically confirmed GBM	mTOR1	Everolimus + TMZ	>18	103	Phase I Trial	Level IV
Migliorini et al	2017	Pleomorphic xanthoastrocytoma	BRAF + MEK	Dabrafenib + Trametinib	Median age 32	1	Case report	Level VI
Rosenberg et al	2022	HGG, GBM, anaplastic gangioglioma, diffuse midline glioma, high-grade neuroepithelial tumor, anaplastic astrocytoma, anaplastic astroblastoma	BRAF; BRAF + MEK	Vemurafenib/Dabrafenib (BRAF) + Trametinib (MEK)	2.3-21.4	19	Multi-institutional Retrospective Analysis	Level IV
Sanai et al	2018	1st recurrence GBM	Wee1K	AZD1775	Median age 59	20	Phase 0 Trial	Level VI
Schiff et al	2015	25 recurrent GBM; 1 Astrocytoma	MET + VEGFR2	Cabozatinib	56.5	26	Phase I trial	Level IV
Shah et al	2007	Various gliomas	PDGFR	Imatinib + Hydroxyurea	Median age 47	16	Retrospective Review	Level IV
Shi et al	2019	BRAF V600E IDH-wt, 1p19q co-deletion Glioma	BRAF V600E	Vemurafenib and Everolimus	Median age 22	1	Case Report	Level VI
Werner et al	2022	Grade III and IV treatment-refractory gliomas	Multitarget kinase	Regorafenib	Median age 54	30	Phase II Trial	Level IV
Wick et al	2019	IDH-wt, MGMT-negative GBM	ALK mTOR MDM2 SHH CDK4/6	Alectinib Temsirolimus Idasanutlin Vismodegib Palbociclib	Still Recruiting Patients		Open-label, Multicenter, Phase I/IIa Trial	Level II

Yau et al	2020	Grade I Unresectable BRAF V600E ganglioglioma	BRAF + MEK	Vemurafenib and Cobimetinib	Median age 32	1	Case Report	Level VI
Zustovich et al	2013	Histologically confirmed GBM	Multitarget kinase	Sorafenib	Median age 60	43	Phase II Trial	Level IV
**Microenvironmental Targets (angiogenesis, cell-cell adhesion, iron/cation regulation)**								
Badruddoja et al	2017	Recurrent GBM	VEGF	Bevacizumab + TMZ	55.00 ± 14.81 years	26	Phase II Trial	Level II
Brown et al	2016	GBM with prior resection, radio, chemo, and TMZ	VEGFR + EGFR	Cediranib + Gefitinib/placebo	30-71	38	Phase II Trial	Level IV
Clarke et al	2014	Newly diagnosed, surgically confirmed GBM or gliosarcoma, with study treatment starting 3–5 wk after open surgery or 2–5 wk after biopsy	VEGF + tyrosine kinase	Bevacizumab + Erlotinib	Median age 54	59	Phase II Trial	Level IV
D'Alessandris et al	2013	Recurrent GBM	VEGF + EGFRvIII	Bevacizumab + Erlotinib	30-77	10	Prospective Trial	Level IV
Desjardins et al	2012	Histologically documented GBM	VEGF	Bevacizumab	Median age 56	32	Phase II Trial	Level IV
Hasselbalch et al	2010	Grade IV astrocytoma/GBM	EGFR, VEGF, topoisomerase I	Cetuximab + Bevacizumab + Irinotecan	50.5–57.9	61	Phase II Trial	Level IV
Lassen et al	2015	Recurrent GBM	Placental growth factor (PlGF) + VEGF	RO5323441 + Bevacizumab	58	22	Phase Ib Trial	Level II
Lu et al	2014	GBM and anaplastic astrocytoma	VEGF	Bevacizumab + TMZ	Not Provided	15	Clinical Trial	Level IV
Prados et al	2009	Newly diagnosed GBM or gliosarcoma	EGFR	Erlotinib + TMZ + RT	Median age 55	65	Phase II Trial	Level IV
Vaccaro et al	2014	Recurrent gliomas of various types	VEGF	Bevacizumab	Median age 38	26	Phase I Trial	Level IV
Vredenburgh et al	2012	Newly diagnosed GBM	VEGF	Bevacizumab + RT + TMZ	56.2	125	Phase I Trial	Level IV

Wang et al	2014	GBM	EGFR	Nimotuzumab, TMZ and RT	Median age 50	26	Phase I Trial	Level IV
Wang et al	2017	Recurrent gliomas	VEGFR2	Apatinib + Irinotecan	Median age 49	10	Pilot Study	Level IV
Weller et al	2017	EGFRvIII GBM	EGFR	TMZ +/- Rindopepimut	Median ages 58,59	338	Phase III Trial	Level II
Wick et al	2020	GBM or oligodendroma	TGF B	TMZ+RT +/- Galunisertib	58.4	33	Phase II Trial	Level II
**Immunotherapy Pathways**								
Anghileri et al	2021	Lynch Syndrome recurrent GBM	PD1	Nivolumab	Median age 33	1	Case Report	Level VI
Nayak et al	2021	Recurrent GBM (bevac naive)	PD1 + VEGF	Pembrolizumab + Bevacizumab	Median age 53	80	Phase II Trial	Level IV
Reardon et al	2020	Recurrent GBM treated with TMZ and radiation originally	PD1	Nivolumab	55.5	184	Phase III Trial	Level II
**Cell Cycle/Apoptosis/Transcription Pathways**								
Brachman et al	2015	Newly diagnosed, supratentorial, histologically confirmed GBM or gliosarcoma	Thioredoxin reductase + ribonucleotide reductase	Motexafin Gadolinium + TMZ + RT	Not Provided	Phase 1 21; Phase 2 92	Phase I/II Trial	Level II
Kubicek et al	2009	17 GBM, 1 anaplastic oligodendroglioma, 1 astrocytoma	26S Proteasome	Bortezomib	Median age 52	23	Phase I Trial	Level IV
Lin et al	2020	Spinal astrocytoma	CDK4	Palbociclib	Median age 38	1	Case Report	Level VI
**Other**								
Desjardins et al	2011	GBM, WHO grade IV	Farnesyl transferase	SCH 66336	Median age 51	36	Phase I Trial	Level IV
Geletneky et al	2017	GBM	Protein NS1	Rat H-1 parvovirus (H-1PV)	57.8 +/- 10.6	18	Phase I/IIa Trial	Level IV
Hashimoto et al	2015	GBM confirmed by pathological review and IDH-wt status	WT1 (Wilms Tumor 1)	WT1 peptide vaccination + TMZ	Median age 49	7	Phase I Trial	Level IV
Patel et al	2012	Grade III and IV glioma	ER	Tamoxifen + TMZ + RT	Median age 51	17	Phase I Trial	Level IV
Sauter et al	2022	Primary inoperable or recurrent GBM	CSF1R, ABL, cKIT, PDGFR	Imatinib	63,52 (median for each arm)	51	Phase II Trial	Level IV

Abbreviation: RCT, randomized control trial; DIPG, diffuse intrinsic pontine glioma; EGFR, epidermal growth factor receptor; GBM, glioblastoma multiforme; PFS, progression-free survival; PT, patient; RR, response rate; VEGFR, vascular endothelial growth factor; CDK, cyclin-dependent kinase; EGFR,

epidermal growth factor receptor; GBM, glioblastoma multiforme; HGG, high grade glioma; IDH, isocitrate dehydrogenase; PDGFR, platelet-derived growth factor receptor; TMZ, temozolomide; VEGFR, vascular endothelial growth factor receptor; WHO, World Health Organization

*Age variables include the provided averages or range from each respective paper, unless otherwise stated as a median age

Supplementary Table S2. Detailed Study Design of Laboratory Studies Implementing Molecular Targeted Therapies in Glioma

Study Author	Year	Tumor Sample	Molecular target	Intervention	Design	3D Culture	Study Subject	Modality
**Protein Kinase Pathways**								
Aldea et al	2014	Patient Samples	mTOR + RAF	Metformin + Sorafenib	in vitro	Yes	Human cell line	small molecule
Aoki et al	2013	C6	Ras	Nobiletin	in vivo	No	Rat cell line	citrus flavonoid
Arcella et al.	2013	U87	mTOR	Rapamycin	in vivo and ex vivo	No	Human cell line	macrolide
Ariey-Bonnet et al.	2020	U87, U87vIII, T98, U251	MAPK14	Benzimidazole	in vitro and in silico	Yes	Human cell line	anthelmintic small molecule
Balkhi et al	2016	C6	Multitarget kinases	Caffeic Acid Phenethyl Ester (CAPE) + Dasatinib	in vitro	No	Rat cell line	small molecule
Barbarisi et al.	2018	T98, A172	CD44	Quercetin, Temozolomide	in vitro	No	Human cell line	nanocarriers
Benezra et al	2012	murine GBM	Multitarget kinases	Dasatanib	in vitro and in vivo	No	Mouse cell line	small molecule
Camorani et al.	2015	U87vIII	EGFRvIII	CL4 aptamer with EGFR TKIs	in vitro	No	Human cell line	aptamer
Chen et al.	2019	U87, U118, A172, LN18	CD163 pathway (CK2, kinase)	TBB (4,5,6,7-tetrabromo-1H-benzotriazole)	in vitro and in vivo	Yes	Human cell line	small molecule
Cheng et al.	2022	LN229, T98, A172	CTSC (Cysteine cathepsin C)	Piperlongumine, Scopoletin	in vitro	No	Human cell line	small molecule
Ciesielski et al	2018	U87 and T98G	Src-kinase + tubulin polymerization inhibitory activity	KX2-361	in vivo	No	Mice	small molecule
Cloninger et al	2011	U87 and LN229	SAPK2/p38 + mTORC1	SB203580 + Rapamycin	in vitro and in vivo	No	Mice	small molecule

Combs et al	2007	U87, LN229, LN18, NCH 82, and NCH 89	EGFR	Cetuximab	in vitro	No	Human cell line	Monoclonal antibody
Dasgupta et al	2015	AM-38 and DBTRG05 MG	BRAF V600E	PLX4720 + RT	in vitro and in vivo	No	Human cell line	small molecule
Dantas-Barbosa et al.	2015	U87, U118, ependymoma, IGRG121 glioma	NOTCH, FBXW7 downregulation	γ-secretase inhibitor RO4929097	in vitro and in vivo	No	Human cell lines	small molecule
Davare et al.	2018	U118, Ba/F3, NIH3T3	ROS1	Lorlatinib	in vitro and in vivo	Yes	Human cell line	small molecule
Di Stefano et al.	2015	GIC-1123	FGFR kinase	JNJ-42756493	in vitro and in vivo	Yes	GIC	small molecule
Dominguez et al.	2013	U87, U251, patient samples	DGKalpha	R59022, R59949	in vitro and in vivo	No	Human cell line, Rat	kinase inhibitor and siRNA
Du et al	2012	BT325 and U251	Raf/MEK/ERK signaling pathway	Sorafenib + Vitamin K	in vitro	No	Human cell line	small molecule
Emlet et al.	2014	patient samples	EGFRvIII + CD133	EGFRvIII, CD133 AB	in vivo	Yes	Human tumor cell line, mice	bispecific antibody
Farrell et al	2017	U87	MET	WO2010/019899A1, PF04217903, Crizotinib	in vivo	No	Mice and Human cell line	small molecule
Feng et al.	2010	C6 rat glioma (IDH-wt)	PI3K/Akt; JNK; ERK	Tamoxifen	in vitro	No	Rat cell line	estrogen receptor inhibitor
Glassman et al	2021	A172, A1207, U87MG, U178MG, U251MG, U373MG, SK-MG4, SK-MG5, SK-MG15, TC620, C6 rat cell line	MAPK kinase	U0126	in vitro	No	Human and rat cell lines	small molecule
Goker et al	2020	T98G	ALK	AZD3463 + TMZ	in vitro	No	Human cell line	small molecule
Golubovskaya et al	2013	U87	FAK	Y15	in vitro and in vivo	No	Human cell line	small molecule

Grossauer et al.	2016	BRAFV600E, Glioma cells	BRAF/MEK	Dabrafenib, Trametinib	in vitro and in vivo	No	Mouse cell line	small molecule inhibitors
Gursel et al.	2011	Mouse and human astrocytomas	PI3K/Akt	PI103	in vivo	No	Human and mouse glioma cell lines transplanted into mice	kinase inhibitor
He et al	2016	U251 and U87	MEK2	MEK2 antibody	in vitro and in vivo	No	Human cell line	Antibody
Hjelmeland et al	2007	U87 and U373	Raf + TOR	LBT613 + Everolimus	in vitro and in vivo	No	Mice and Human cell line	small molecule
Hong et al	2014	Patient Samples	Aurora-A kinase	Alisertib	in vitro	Yes	Human cell line	small molecule
Jiang et al.	2018	U87, U251	EGFR/EGFRvIII	EGFR/EGFRvIII CAR T cells	in vitro and in vivo	No	Human cell line	chimeric antigen receptor T cells
Jin et al.	2013	U87, U251	Notch + AKT	MRK003, MK-2206	in vitro	No	Human cell line	kinase inhibitor
Joel et al.	2015	GICs from T08	PBK/TOPK	HI-TOPK-032	in vitro and in vivo	Yes	Patient derived GIC cultures, T08 cells into mice	small molecule
Joshi et al	2012	GBM oncosphere line 020913	Multitarget kinases	Gefitinib, Erlotinib, Sunitinib	in vitro and in vivo	Yes	Mice and Human cell line	small molecule
Ju et al	2016	U87	COX-2	Celecoxib	in vitro and in vivo	Yes	Mice and Human cell line	small molecule
Junca et al.	2017	patient samples	ALK, ROS1, MET	Crizotinib	in vitro	Yes	Human tumor cells	small molecule
Jung et al.	2014	U87, U251	FOXO3A	Z-ajoene	in vitro	Yes	Human cell line	garlic derived molecule
Kawauchi et al	2021	U87, LN229, and GSC23	ALK	Alectinib + Ceritinib	in vitro and in vivo	Yes	Mice and Human cell line	small molecule
Kim et al	2012	U87 and Patient Samples	Phosphoinositide 3-kinase/AKT and Ras/Raf	5-Bromo-3-(3-hydroxyprop-1-ynyl)-2H-pyran-2-one (BHP)	in vitro and in vivo	Yes	Mice and Human cell line	small molecule
Koul et al.	2005	U87, U251, LN229,	Integrin-linked kinase	QLT0276	in vitro	No	Human cell line	kinase inhibitor

		SNB19, U373, D54						
Koul et al.	2010	U87	PI3K/AKT	PX-866	in vitro and in vivo	No	Human tumor cell lines and mouse xenografts	kinase inhibitor
Liu et al.	2011	U251	basic Fibroblast Growth Factor (bFGF) - STAT3 pathway	Anti bFGF siRNA	in vitro	No	Human cell line	siRNA
Liu et al.	2014	U87	EGFR & PI3K/AKT	G19	in vitro and in vivo	No	Human cell line	oligosaccharide
Liu et al	2014	T98G, A172 and U87	AMPK	Compound C	in vitro	Yes	Human cell line	small molecule
Luchman et al	2014	Patient sample	mTOR1/2	AZD8055	In vitro and in Vivo	Yes	Human cell line and mice	small molecule
Ma et al.	2015	U87, U251	STAT3	Tetrandrine	in vivo	No	Human cell line and tumor cells	alkaloid
Matsuda et al	2012	U87, T98G, patient samples	JNK	SP600125	In vitro and in Vivo	Yes	Human cell line and mice	small molecule
Maxwell et al	2021	BT40	mTOR1/2 + MEK	TAK228 + Trametinib	In vivo	No	Mice	small molecule
Nicolaides et al	2011	AM38	BRAF	PLX4720	In vivo	No	Mice	small molecule
Paternot et al	2009	T98G, U87MG, U138MG	mTOR1 + MEK1/2	Rapamycin + PD184352	In vitro	No	Human cell line	small molecule
Peng et al.	2013	U87, CHG5	RACK1-PKC	siRNA	in vitro and in vivo	No	Human cell and tumor lines	siRNA
Pezuk et al	2013	U251, U138, U87, T98G, U343 and MO59K	PLK1	BI2536 + TMZ	in vitro	No	Human cell line	small molecule
Phillips et al.	2016	A431, U87	EGFR	ABT-414	in vitro and in vivo	No	Human cell line	antibody-small molecule fusion
Premkumar et al	2010	U87, T98G, U373, LN229 and	IGF1R + Src	NVP-AEW541 + Dasatinib		No	Human cell line	small molecule

		A172						
Qin et al.	2014	U87, U138, U373	EMP2	Anti-EMP2 antibodies, Anti-EMP2 IgG1	in vitro and in vivo	No	Human cell line	antibody
Raub et al	2015	U87	CDK4 + CDK6	Abemaciclib or Palbociclib + TMZ	In vitro and in vivo	No	Human cell line and rats	small molecule
Salphati et al.	2012	U87, GS2, GBM10	PI3k	GNE-317	in vitro and in vivo	Yes	Human cell line in mice	kinase inhibitor
Sathornsumetee et al	2006	U87MG, T98G, and U373MG	BRAF, CRAF, VEGFR	AAL881	In vitro and in vivo	No	Human cell line and mice	small molecule
See et al	2012	19 different GBM lines	MEK + PI3K/mTOR	Vemurafenib + PI103	In vitro and in vivo	No	Human cell lines	small molecule
Selvasaravanan et al	2020	U87GM and A172	MEK or PI3K	Trametinib + Pictilisib	In vitro	Yes	Human cell lines	small molecule
Shingu et al	2015	U87MG, LNZ308, LN428	MEK, EGFR, PI3K	Various small molecule inhibitors	in vitro	Yes	Human cell lines	small molecule
Siegelin et al	2010	U87, U251, LN229	BRAF	Sorafenib	In vitro and in vivo	No	Human cell lines and mice	small molecule
Signore et al.	2014	U87, T98	PDK1 + CHK1	UCN-01	in vitro and in vivo	No	Human cell line	staurosporin derivative
Spino et al	2019	Patient sample	DLL3	Rovalpituzumab tesirine	In vitro	Yes	Human cell lines	Monoclonal antibody
Thanasupawat et al	2017	U87 and U251	FGFR	Dovitinib	In vitro and in vivo	No	Human cell lines and mice	small molecule
Thompson et al	2018	Patient sample	Various	Various antibodies + kinase inhibitors + chemo drugs	In vivo	No	Mice	Small molecule and MAB
Tsigelny et al	2017	U87 and patient sample	OLIG2	SKOG102	In vitro and in vivo	Yes	Human cell lines	small molecule
van den Heufel	2017	E98	MET	Compound A	In vitro and in vivo	Yes	Human cell lines and mice	small molecule
Wang et al	2013	U87, U251, and patient samples	MEK1	miR-181b + TMZ	In vitro	No	Human cell lines	miRNA
Wang et al.	2014	U87, U251	RAS	miR-143	in vitro and in vivo	No	Human cell line and tumor cells	miRNA

Wang et al.	2019	Glioma stem cells from patient xenografts	EGFR or PI3K w/ DHODH	Lapatinib (EGFR) + BKM120 (PI3K) + Teriflunomide	in vitro and in vivo	No	Human cell line	small molecule
Wichmann et al.	2015	U251, LN229	EGFR + HER2	siRNAs + therapeutic antibodies (EGFR: Cetuximab; HER2: Trastuzumab)	in vitro	No	Human cell lines	siRNA, small molecule
Yan et al	2017	DF1-virus laden cells	CSF-1R + cKIT + RTKs	PLX3397 + Vatalanib + Dovitinib	In vivo	No	Mice	Small molecule
Yang et al	2008	F98	EGFR	Boronated EGFR MAB + Cetuximab	In vitro and in vivo	No	Rats	Small molecule and MAB
Yao et al.	2015	AM38, DBTRG-05MG, NMC-G1	EGFR + BRAF	BRAF(V600E) inhibitor PLX4720	in vitro and in vivo	No	Human cell lines, orthotopic mouse model	small molecule
Zavalhia et al	2014	Patient sample	cKIT	Imatinib	ex vivo	No	Human tumors	Small molecules
Zhang et al.	2015	U87	mGluR1	siRNA ,Riluzole , BAY36-7620	in vitro and in vivo	No	U87, U87 orthotopic mouse model	siRNA, small molecule
Zhang et al.	2016	patient samples	HER2	HER2 specific NK cells	in vitro and in vivo	Yes	Human tumor cells and cell lines	modified cells
Zhang et al	2017	AM-38, DBTRG-05MG, NMC-G1	BRAF V600E + MEK	PLX4032 + GDC0973	In vivo	No	Mice	Small molecule
**Cell Cycle/Apoptosis/Transcription Pathways**								
Bychov et al.	2020	U251, A172	ASIC1a containing channels	Mambalgin-2	in vitro	Yes	Human cell line	venom derivative
Chen et al.	2013	U251, SHG44	S100A9 (a heterodimer for calprotectin)	shRNA	in vivo and ex vivo	Yes	Human cell lines in mice and tumor cells	shRNA in virus
Chen et al.	2019	U87, U118	IGFBP3	IGFBP3 siRNA	in vitro and in vivo	No	Human cell line	siRNA
Grinshtein et al.	2016	patient samples	HDAC/EZH2	Compound 26/unc1999	in vitro and in vivo	Yes	Human cell line	small molecules

Festa et al.	2011	patient samples	BAG3	BAG3 siRNA	in vitro and in vivo	No	Rats with human transplants	siRNA
Ge et al.	2013	U87	miR-27a (FOXO3a)	AntagomiR-27a	in vitro and in vivo	No	Human cell line	miRNA inhibitor
Genoud et al	2021	SB28 and GL261	Tumor checkpoint controller targeting microtubules	BAL101553	in vivo and ex vivo	No	Mouse cell line	small molecule
Gu et al.	2015	U87, SHG44, CHG5, U251	PAK5	PAK5 shRNA	in vitro and in vivo	No	human cell line and tumor cells	shRNA
Guo et al	2011	U87	DR4/5	TRAIL + Doxorubicin	in vitro and in vivo	No	Human cell line	small molecule
Hamada et al.	2022	HEK293T	CDK 4/6 + PDGFRα	Lenvatinb, Crernolanib, Abemaciclib, Palbociclib	in vitro	Yes	Human cell line	small molecules
Joshi et al.	2017	U87, D54	Procaspase-3	PAC-1 (*activating molecule)	in vitro and in vivo	Yes	Human and rat tumor cells	small molecule
Kalluri et al.	2017	GSCs from gliomas	Phospholipase C	D609	in vitro	Yes	Human tumor cells	small molecule
Kaneta et al.	2013	U1242	NEK9	NEK9-siRNA	in vitro	No	Human cell line	siRNA
Kong et al.	2018	U87, T98	BMI1	PTC-209	in vivo and in vitro	Yes	Human cell line	small molecule
Lamour et al.	2015	U87	Osteopontin	shRNA	in vitro and in vivo	Yes	GICs, orthotopic mouse model	shRNA
Lee et al.	2012	SF188, U251	Polo-like kinase 1 (PLK1)	BI2536	in vitro and in vivo	Yes	Human cell line in mice	competitive kinase inhibitor
Lescarbeau et al.	2016	patient samples	Wee1K	MK-1775	in vitro and in vivo	No	Human tumor cells	small molecule
Li et al.	2012	U87, U251	p53/MDM2	D-PMIBeta	in vitro and in vivo	No	Human cell line in mice	palmylated D-peptide
Lian et al.	2013	SHG44, U251, U87	miR-23a (APAF1)	Anti-miR-23a	ex vivo	No	Human tumor	miRNA inhibitor
Liu et al	2019	U87, U251, U118,	EGFR	AZD9291	in vitro and in	Yes	Human cell line	small molecule

		LN229, T98G and LN18			vivo			
Mao et al.	2013	U87, SNB19, SNB44, SNB75, U118, U563, A172, SNB19	STK17A	Anti-STK17A shRNA	in vitro	No	Human cell line	shRNA
Merlino et al.	2018	U87	MDM2/4 + α5β1/αvβ3	Compound 9	in vitro	No	Human cell line	peptidomimetic
Michaud et al.	2010	patient samples	CDK4/6	PD-0332991	in vitro and in vivo	No	Human tumor cell lines and mouse xenografts	kinase inhibitor
Niu et al.	2015	U87, A172, SHG44, U251	FOXM1	Plumbagin	in vitro an in vivo	No	Human cell lines, human cell lines in mice	natural compound
Nonnenmacher et al.	2015	TiC35 in mice	XIAP + BCL-2	RIST (Rapamycin, Irinotecan, Sunitinib, Temozolomide) + the variant aRIST (alternative to Rapamycin, GDC-0941	in vitro	No	Human cell lines and primary cultured patient material, orthotopic mouse model	small molecule
Patyka et al.	2016	U87, T98, A172, U138 LN18	MGMT	PRIMA-1MET	in vitro	Yes	Human cell lines	small molecule
Punganuru et al.	2020	U87	MDM2	SP-141	in vitro and in vivo	No	Human cell line	small molecule
Sasame et al.	2022	293T	HSP90	BIIB021, 17-AAG (HSP90 inhibitor) + BRAFi + MEKi	in vitro and in vivo	Yes	Human cell line	siRNA
Tasaki et al.	2016	Grade II-IV Gliomas	HGFR aka MET	Crizotinib	in vitro and in vivo	Yes	Human tumor cells	small molecule
Tchoghandjian et al.	2016	patient samples	IAPs	GDC-0152	in vitro and in vivo	Yes	Human tumor cells	small molecule
Vengoji et al	2019	U87	EGFR	Afatinib + TMZ	In vitro and in	Yes	Human cell lines and mice	small molecule

					vivo			
Wang et al.	2011	U87	Survivin	Survivin-siRNA/Transferrin receptor conjugate	in vitro and in vivo	No	Human cell line and rats	siRNA
Wang et al.	2019	U87	EZH2	EZH2si-DMC	in vitro and in vivo	Yes	Human cell line	siRNA
Wang et al	2019	Glioma stem cells	Carbamoyl-phosphate synthetase 2 (CAD)	Teriflunomide	In vitro and in vivo	Yes	Human cell line and mice	siRNA
Xu et al.	2017	A172, T98, HEK293T, MDA-MB-231, T-47D, U87, U138, U251, U343	BCL6	RI-BPI	in vitro and in vivo	No	Human cell lines	peptide
Xu et al.	2020	U87, U251, GSC267	CUL7	miR-3940-5p	in vitro and in vivo	Yes	Human cell line	microRNA
Yang et al	2006	F98	EGFRvIII	L8A4	in vitro and in vivo	No	Rat cell line	Monoclonal antibody
Zhang et al.	2011	T98G, LN229	eEF2-kinase	eEF2-siRNA	in vitro	No	Human cell line	siRNA
Zhao et al.	2015	U87, U251, SHG44	ID2	Anti-ID2 siRNA	in vitro	No	human cell line	siRNA
Zhong et al.	2018	U87, U251, LN18, T98, SHG-44, U373, HUVEC, hepatocyte (HL7702)	CDK + Aurora (dual inhibitor)	JNJ-7706621	in vitro	No	Human cell line	small molecule
**Microenvironmental Targets (angiogenesis, cell-cell adhesion, iron/cation regulation)**								
Abdul Rahim et al.	2017	patient samples	ATG9A	Bevacizumab +/- Chloroquine	in vivo and ex vivo	Yes	Human tumor cells	monoclonal antibody with small molecule
Angara et al	2017	U251	20-HETE	HET0016	in vivo	No	Rats	Small molecule
Blanco et al.	2014	U87vIII	Phosphatidylserine	SAPc-DOPS	in vitro and in vivo	No	human cell line	lysosomal protein
Blank et al.	2001	C6 rat glioblastoma	Endothelial pigpen protein	Aptamer III.1	in vitro	No	Mouse tissue samples	aptamer
Chen et al.	2013	U87, C6	NRP-1	NRP-1 Mab	in vitro and in vivo	No	Human and rat cell line in mice	monoclonal antibody
Fleurence et	2016	patient	O-acetyl GD2	Anti-GD2	in vitro	Yes	Human	monoclonal

al.		samples	ganglioside	antibody	and in vivo		tumor cells and cell lines	antibody
Franco et al.	2018	U87	TFAM	Melatonin	in vitro	No	human cell line	hormone
Grossman et al	2013	U87	Pan-VEGF	Cediranib + TMZ	in vivo	No	Rats	small molecule
He et al.	2018	NSCG cells	LTβR	LIGHT-VTP	in vitro and in vivo	No	human cell line	cytokine/vascular targeting peptide
Huang et al.	2021	U87, LN18, U118	TRPV4	Cannabidiol	in vitro and in vivo	Yes	Human cell line	phytocannabinoid
Huveldt et al.	2013	GBM10	VEGF + Src Family kinases	Bevacizumab, Dasatinib	in vivo	No	Human cell line transplanted into mice	monoclonal antibody with small molecule
Jaszberenyi et al.	2013	U87	Growth-Hormone Releasing Hormone	MIA-604, MIA-690	in vitro and in vivo	No	Human cell line in mice	Hormone analog
Ji et al.	2013	patient samples, U251	Nrf2	siRNA	in vivo and ex vivo	No	Human cell line in mice and human tumor cells	siRNA
Kuan et al.	2010	patient samples	MRP3	Anti-MRP antibody	in vitro and ex vivo	No	Patient samples and human cell line	monoclonal antibody
Lu et al.	2015	U87, MGG4 GSC-derived, 005 GSC-derived	VEGFR	TKI VEGF inhibitor	in vitro and in vivo	Yes	Human and murine cell lines	small molecule
Mojarad-Jabali et al.	2022	GL261 (IDH-wt)	TfR (transferrin receptor)	T12, B6, T7 (TfR-targeting peptides)	in vitro and in vivo	No	Murine cell line	small molecules
Mostafavi et al.	2015	U87, 1321N1	CX43 + miR21	B2 cAMP agonist	in vitro	No	Human cell lines	small molecule
Nandhu et al.	2018	U251	Fibulin-3		in vitro and in vivo	Yes	Human cell line	monoclonal antibody
Nawashiro et al.	2006	C6, patient samples	LAT1	BCH	in vivo and ex vivo	No	Patient samples and rat cell lines	small molecule
Pall et al.	2019	U251, U87	NHE9	Gold nanoparticle-enabled	in vitro	No	Human cell line	nanoparticle

				photothermal therapy (NEPTT)				
Phillips et al.	2019	DIPG-VI, GBM-0401	Lanosterol synthase	MI-2 (menin inhibitor)	in vitro	No	Human cell line	small molecule
Renfrow et al.	2020	patient samples	HIF2α	PT2385	in vitro and in vivo	No	Human cell line	small molecule
Saw et al.	2021	U87, U251, U373, MCF7, PC3, B16F10, B16F1	EDB-FN (extra domain B of fibronectin)	Docetaxel-loaded EDB-FN specific micelles	in vitro and in vivo	Yes	Human cell line	targeted micelle with taxane
Takano et al	2003	U87MG and U251MG	VEGF	Anti-VEGF AB + Nimustine	In vitro and in vivo	No	Human cell line and mice	MAB
Tyrinova et al.	2018	Grade III-IV gliomas	tmTNFa	Recombinant IL2 or dsDNA	ex vivo	No	Patient samples	interleukin or double-stranded DNA
Watanabe et al.	2020	U251	CTL1 (choline transporter-like protein 1)	Amb4269951	in vitro and in vivo	No	Human cell line	small molecule
Xia et al.	2022	U251, U87	VEGFR2	Apatinib	in vitro and in vivo	No	Human cell line	small molecule
Xiong et al.	2019	MCF7, HL60, MCF7	Calmodulin, EGFR, Aromatase	W-13, Gefitinib, Exemestane	in silico	No	miRNA expression profiling in GBM were obtained from the Gene Expression Omnibus (GEO) database	small molecule
Xu et al.	2019	U87, LN229	ITGA9	miR-148a	in vitro and in vivo	No	Human cell line	miRNA
**Immunotherapy Pathways**								
Baehr et al.	2017	U87, LN229, U251, LN308	STING (stimulator of interferon gene)	ASA404	in vitro and in vivo	No	Human cell line	small molecule
Goswami et al.	2020	GL261	CD73	Anti-CD73	in vitro and in vivo	No	Murine cell line	monoclonal antibody
Merrill et al.	2004	G3 anaplastic	CD155 receptor	PVS-RIPO	in vitro	No	Patient samples	recombinant virus

		astrocytoma, G4 GBM						
Schleicher et al.	2011	GL261	ATX + LPA receptors	siRNA	in vitro and in vivo	No	Human cell lines and mice	siRNA
Xu et al	2015	U87	EMMPRIN	Icaritin	in vitro	No	Human cell line	small molecule
Zanotto-Filho et al.	2011	C6 rat and U138, U87, U373	NFkB	BAY117082, MG132	in vitro	No	Rat and human cell lines	small molecule
Zhang et al.	2009	U87, U251, human astrocytoma cells	Formyl Peptide Receptor (FPR)	F2 procyanidins	in vitro	No	Human cell line	Oligomer
**Other Pathways/Targets**								
Barone et al.	2014	U87	CXCR4 + VEGF	POL555, mcr89	in vitro and in vivo	Yes	Human cell line	protein epitope mimetic & antibody
Caruana et al.	2017	T98, U87, A172	Site-1 protease	PF-429242	in vitro	No	Human cell lines	small molecule
Chen et al.	2013	C6	CXCR4	Tetramethylpyrazine	in vitro	No	Rat cell line	small molecule
Chen et al.	2021	U251, U87, SHG44, A172	miR-106a-5p	Circ-ITCH	in vitro and in vivo	No	Patient samples and human cell lines	circRNA
Colen et al.	2011	U87	Lactate (monocarboxylate) transporters	α-cyano-4-hydroxycinnamic acid	in vitro and in vivo	No	Human cell lines, rat	lactate transporter inhibitor
Harford-Wright et al.	2017	U87, LN229	Apelin G-protein coupled receptor	MM54, MM193	in vitro and in vivo	Yes	Human tumor cells	small molecule
Ishiwata et al.	2011	patient samples, A172 GBM, KG-1-C glioma	Nestin	Anti-Nestin IgG	in vitro and ex vivo	Yes	Human cell line and patient samples	Immunoglobulin
Jiang et al.	2021	Gliomas from GSE31095 and GSE109857 from the NCBI Gene Expression Comprehensive database	EEF1A1 + RPL11	Puromycin, Doxorubicin, Daunorubicin, Mitoxantrone	ex vivo	No	Patient samples	Small molecules
Kim et al.	2018	U87, T98, LN18	MALAT1	siRNA, Temozolomide	in vitro and in vivo	Yes	Human cell line	siRNA

Kim et al.	2019	U87, U373	IDH1R132H	AGI-5198 (in combo with HDACi)	in vitro	No	Human cell line	small molecule
Li et al.	2018	U251, U87, non-glioma human tumor cells	hnRNP A1/B2	β-Asarone	in vitro	No	Human tumor cells	plant product
Liu et al.	2016	U251, SHG-44	CRM1	S109	in vitro and in vivo	No	Patient samples and human cell line	small molecule
Loskutov et al.	2018	patient samples	LPAR1/3	Ki16425	in vivo and in vitro	Yes	Human tumor cells	small molecule
Luwor et al.	2019	U87, U251	Dynamin 2	Dynole 34-2, CyDyn 4-36	in vitro and in vivo	Yes	Human cell lines	
Miyazaki et al.	2012	8 human GBM cell lines	c-Myb	Telomestatin	in vitro and in vivo	Yes	Human cell lines	hydrophobic agent
Peng et al.	2019	U373, U87, U251	miR-25	miR-25 inhibitor	in vitro	No	Human cell line	miRNA
Piunti et al.	2017	SF8628, SF7761, SU-DIPG-IV, pcGBM2, SF9402, SF9427	PRC2 + BET bromodomain proteins	JQ1, I-BET	in vitro and in vivo	No	Human cell lines	small molecule
Preukschas et al.	2012	G55T2, U87, U251	eIF-5A, DHS, DOHH (both eIF-5A activators)	GC7	in vitro	No	Human cell line	small molecule
Saito et al	2004	U87	TRAILR	Recombinant TRAIL + TMZ	In vitro and in vivo	No	Human cell line and rats	Recombinant human protein
Saito et al.	2014	U87, U251, KNS81, T98G, SF126, KALS-1	EFTUD1	EFTUD1 shRNA	in vitro and in vivo	No	Human cell line and patient samples	shRNA
Sanzey et al.	2015	U87, U251, T98	PFK1	Clotrimazole	in vitro and in vivo	Yes	Patient samples	small molecule
Saunders et al.	2021	GBM39, GBM43	YAP1 (yes-associated protein 1)	NSC682769	in vitro and in vivo	No	Human cell line	small molecule
Shulepko et al.	2020	U251, A172	α7 nAChR	rSLURP-1	in vitro	No	Human cell line	recombinant analogue of human protein
Song et al.	2019	U87, U251,	A1CF +	shRNA	in vitro	No	Human	shRNA

		HEK293T	FAM224A		and in vivo		cell line	
Spino et al.	2019	U87, MGG18, MGG152, MGG119	DLL3 (Notch ligand)	Rova-T (Rovalpituzumab tesirine)	in vitro	Yes	Human cell line	antibody–drug conjugate (ADC)
Tu et al.	2017	U87, A172, U251, C6	smoothened	GDC-0449	in vitro and in vivo	No	Human and murine cell lines	small molecule
Venere et al.	2015	patient samples	KIF11	Ispinesib	in vitro and in vivo	Yes	patient samples	small molecule
von Spreckelsen et al.	2021	GBM6	Brevican	Anti-deglycosylated brevican peptide	in vitro and in vivo	No	Human cell line	peptide
Wu et al.	2011	U251, T98, A172	miR-128	Ginsenoside Rh2	in vitro	No	Human cell line	small molecule
Yan et al.	2013	U87, SF295	14-3-3	siRNA	in vitro	No	Human cell line	
Zhang et al.	2021	U87	IDH1R132H	WM17	in vitro	No	Human cell line	small molecule
Zhang et al.	2022	U87, U251, A172, LN229, U118, P3	Fat mass + obesity associated protein (FTO)	SPI1 inhibitor DB2313	in vitro and in vivo	Yes	Human cell line	microRNA

Abbreviations: CDK, cyclin-dependent kinase; EGFR, epidermal growth factor receptor; GBM, glioblastoma multiforme; HIF, hypoxia-induced factor; IDH, isocitrate dehydrogenase; miR(NA), micro ribonucleic acid; PDGFR, platelet-derived growth factor receptor; siRNA, small interfering ribonucleic acid; TKI, tyrosine kinase inhibitor; TMZ, temozolomide; TNF, tumor necrosis factor; VEGFR, vascular endothelial growth factor receptor; oligodendroglioma, IDH-mutant/p19q co-deleted Glioma; 20-HETE, 20-hydroxy eicosatetraenoic acid; HGF, hepatocyte growth factor; TRAIL, Tumor necrosis factor-related apoptosis-inducing ligand; ALK, anaplastic lymphoma kinase; ROCK, Rho-associated protein kinase

Supplementary Table S3. Detailed Study Design of Ongoing Clinical Trials on ClinicalTrials.gov Implementing Molecular Targeted Therapies in Glioma

Title	Sponsor/ Collaborator	Funding	Phase	Status	Results (if applicable)
**Protein Kinase Pathways**					
Imatinib Mesylate in Treating Patients With Recurrent Malignant Glioma or Meningioma	Sidney Kimmel Comprehensive Cancer Center at Johns Hopkins| NCI	NIH	Phase 1| Phase 2	Completed	Six-month progression-free survival was 3% for glioblastoma multiforme and 10% for anaplastic glioma patients. CYP3A4 inducers, such as enzyme-inducing antiepileptic drugs, substantially decreased plasma exposure of imatinib
Gefitinib in Treating Patients With Newly Diagnosed Glioblastoma Multiforme	National Cancer Institute (NCI)	NIH	Phase 2	Completed	
CCI-779 in Treating	National Cancer Institute	NIH	Phase	Complet	

Patients With Recurrent Glioblastoma Multiforme	(NCI)		2	ed	
Gefitinib in Treating Patients With Recurrent or Progressive CNS Tumors	Sidney Kimmel Comprehensive Cancer Center at Johns Hopkins|National Cancer Institute (NCI)	NIH	Phase 2	Complet ed	
Erlotinib in Treating Patients With Solid Tumors and Liver or Kidney Dysfunction	National Cancer Institute (NCI)	NIH	Phase 1	Complet ed	
Gefitinib and Radiation Therapy in Treating Patients With Glioblastoma Multiforme	National Cancer Institute (NCI)|NRG Oncology	NIH	Phase 1| Phase 2	Complet ed	
Imatinib Mesylate in Treating Patients With Gliomas	European Organisation for Research and Treatment of Cancer - EORTC	Other	Phase 2	Complet ed	
Erlotinib in Treating Patients With Recurrent Malignant Glioma or Recurrent or Progressive Meningioma	National Cancer Institute (NCI)	NIH	Phase 1| Phase 2	Complet ed	MTD was 650 mg
Erlotinib and Temozolomide With Radiation Therapy in Treating Patients With Glioblastoma Multiforme or Other Brain Tumors	National Cancer Institute (NCI)	NIH	Phase 2	Complet ed	
A Phase II Exploratory, Multicentre, Open-label, Non-comparative Study of ZD1839 (Iressa) and Radiotherapy in the Treatment of Patients With Glioblastoma Multiforme	AstraZeneca	Industry	Phase 2	Complet ed	
Imatinib Mesylate in Treating Patients With Recurrent Brain Tumor	Alliance for Clinical Trials in Oncology|National Cancer Institute (NCI)	NIH	Phase 1| Phase 2	Complet ed	PFS rates were between 25 and 33.5% depending on EIACs and number of previous regimens
Everolimus and Gefitinib in Treating Patients With Progressive Glioblastoma Multiforme or	Memorial Sloan Kettering Cancer Center|National Cancer Institute (NCI)	NIH	Phase 1| Phase 2	Complet ed	Primary outcome was overall objective response. Partial response in 2/43 patients at highest dose of 70mg everolimus. Remaining patients had stable disease or

Progressive Metastatic Prostate Cancer					progression of disease.
Erlotinib Compared With Temozolomide or Carmustine in Treating Patients With Recurrent Glioblastoma Multiforme	European Organisation for Research and Treatment of Cancer - EORTC	Other	Phase 2	Completed	
Sorafenib in Treating Patients With Recurrent or Progressive Malignant Glioma	National Cancer Institute (NCI)	NIH	Phase 1	Completed	
Lapatinib in Treating Patients With Recurrent Glioblastoma Multiforme	National Cancer Institute (NCI)|NCIC Clinical Trials Group	NIH	Phase 1 |Phase 2	Completed	
GW572016 to Treat Recurrent Malignant Brain Tumors	National Cancer Institute (NCI)|National Institutes of Health Clinical Center (CC)	NIH	Phase 2	Completed	
Ph I Gleevec in Combo w RAD001 + Hydroxyurea for Pts w Recurrent MG	Annick Desjardins|Novartis Pharmaceuticals|Duke University	Industry	Phase 1	Completed	
Phase II Imatinib + Hydroxyurea in Treatment of Patients With Recurrent/Progressive Grade II Low-Grade Glioma (LGG)	Duke University|Novartis Pharmaceuticals	Industry	Phase 2	Completed	Astrocytomas had43.8% PFS and oligodendromas had 34.4% PFS at 12 months
Oral Tarceva Study for Recurrent/Residual Glioblastoma Multiforme and Anaplastic Astrocytoma	Northwell Health|Genentech, Inc.	Industry	Phase 1|Phase 2	Completed	Coexpression of EGFR and PTEN is related to response to EGFR inhibitors
Sorafenib Tosylate and Temsirolimus in Treating Patients With Recurrent Glioblastoma	National Cancer Institute (NCI)	NIH	Phase 1|Phase 2	Completed	Limited activity of sorafenib and temsirolimus in this dose and schedule was observed with considerable grade 3+ toxicity.
Sorafenib Combined With Erlotinib, Tipifarnib, or Temsirolimus in Treating Patients With Recurrent Glioblastoma Multiforme or	National Cancer Institute (NCI)	NIH	Phase 1|Phase 2	Completed	Sorafenib + erlotinib had 42.1%, Sorafenib + temsirolimus had 55.6% 12-mo survival, Sorafenib + tipifarnib combo was too toxic

Gliosarcoma					
Temsirolimus, Temozolomide, and Radiation Therapy in Treating Patients With Newly Diagnosed Glioblastoma Multiforme	National Cancer Institute (NCI)	NIH	Phase 1	Completed	
Tumor Tissue Analysis in Patients Receiving Imatinib Mesylate for Malignant Glioma	Sidney Kimmel Comprehensive Cancer Center at Johns Hopkins|National Cancer Institute (NCI)	NIH	Phase 1	Completed	
Erlotinib and Sorafenib in Treating Patients With Progressive or Recurrent Glioblastoma Multiforme	National Cancer Institute (NCI)	NIH	Phase 2	Completed	14% PFS with no adverse effects reported
Dasatinib in Treating Patients With Recurrent Glioblastoma Multiforme or Gliosarcoma	National Cancer Institute (NCI)|Radiation Therapy Oncology Group|NRG Oncology	NIH	Phase 2	Completed	Dasatinib is safe but ineffective in recurrent GBM
A Phase II Trial of Sutent (Sunitinib; SU011248) for Recurrent Anaplastic Astrocytoma and Glioblastoma	H. Lee Moffitt Cancer Center and Research Institute|Pfizer	Industry	Phase 2	Completed	4/30 patients had 6 month PFS after sunitinib treatment
Ph II Erlotinib + Sirolimus for Pts w Recurrent Malignant Glioma Multiforme	Duke University|Genentech, Inc.|OSI Pharmaceuticals	Industry	Phase 2	Completed	3.1% PFS6, with median PFS 6.9 weeks
Radiation Therapy and Temozolomide Followed by Temozolomide Plus Sorafenib for Glioblastoma Multiforme	SCRI Development Innovations, LLC|Bayer	Industry	Phase 2	Completed	Addition of sorafenib showed no benefit over the standard therapy
Sunitinib Tumor Levels in Patients Not on Enzyme-Inducing Anti-Epileptic Drugs Undergoing Debulking Surgery for Recurrent Glioblastoma	Massachusetts General Hospital|Brigham and Women's Hospital|Dana-Farber Cancer Institute|Pfizer	Industry	Early Phase 1	Completed	
Sunitinib in Treating	National Cancer Institute	NIH	Phase	Complet	1/21 patients with PFS at 6

Patients With Recurrent Malignant Gliomas	(NCI)		2	ed	months
Ph. 2 Sorafenib + Protracted Temozolomide in Recurrent GBM	Duke University|Bayer|Schering-Plough	Industry	Phase 2	Completed	Sorafenib + TMZ is safe but ineffective in recurrent GBM
Ph I Dasatinib + Erlotinib in Recurrent MG	Duke University|Bristol-Myers Squibb|Genentech, Inc.	Industry	Phase 1	Completed	
Ph I SU011248 + Irinotecan in Treatment of Pts w MG	Duke University|Pfizer	Industry	Phase 1	Completed	
BIBW 2992 (Afatinib) With or Without Daily Temozolomide in the Treatment of Patients With Recurrent Malignant Glioma	Boehringer Ingelheim	Industry	Phase 2	Completed	Afatinib is more effective than TMZ alone as monotherapy, though not as combination with TMZ
A Study of Temsirolimus and Bevacizumab in Recurrent Glioblastoma Multiforme	Rigshospitalet, Denmark|University of Copenhagen|Wyeth is now a wholly owned subsidiary of Pfizer|Roche, Copenhagen	Industry	Phase 2	Completed	Temsirolimus can be safely administered in combination with bevacizumab. This study failed to detect activity of such a combination in patients with progressive GBM beyond bevacizumab therapy.
Everolimus in Treating Patients With Recurrent Low-Grade Glioma	Susan Chang|Novartis|University of California, San Francisco	Industry	Phase 2	Completed	Primary outcome was progression free survival at 6 months. 39/47 patients with grade II glioma met 6 month PFS. 6/11 patients with Grade III/IV glioma met 6 month PFS.
Sorafenib in Newly Diagnosed High Grade Glioma	University Hospital, Geneva|Bayer	Industry	Phase 1	Completed	
Everolimus, Temozolomide, and Radiation Therapy in Treating Patients With Newly Diagnosed Glioblastoma	Alliance for Clinical Trials in Oncology|National Cancer Institute (NCI)	NIH	Phase 1|Phase 2	Completed	64% of patients achieved PFS12
Study of Sunitinib Before and During Radiotherapy in Newly Diagnosed Biopsy-only Glioblastoma Patients		Other	Phase 2	Completed	
Dasatinib or	Alliance for Clinical Trials	NIH|Indus	Phase	Complet	The addition of dasatinib to

Placebo, Radiation Therapy, and Temozolomide in Treating Patients With Newly Diagnosed Glioblastoma Multiforme	in Oncology|National Cancer Institute (NCI)|Bristol-Myers Squibb	try	1|Phase 2	ed	conventional therapy did not confer any survival benefit
Open Label Trial to Explore Safety of Combining Afatinib (BIBW 2992) and Radiotherapy With or Without Temozolomide in Newly Diagnosed Glioblastoma Multiform	Boehringer Ingelheim	Industry	Phase 1	Completed	MTD of afatinib was 30 mg for RT + TMZ, and 40mg for RT alone
Radiation Therapy and Temsirolimus or Temozolomide in Treating Patients With Newly Diagnosed Glioblastoma	European Organisation for Research and Treatment of Cancer - EORTC|Pfizer	Industry	Phase 2	Completed	
Temsirolimus and Perifosine in Treating Patients With Recurrent or Progressive Malignant Glioma	National Cancer Institute (NCI)	NIH	Phase 1|Phase 2	Completed	MTD of temsirolimus was 115mg/wk
A Study in Subjects With Recurrent Malignant Glioma	Eisai Inc.	Industry	Phase 2	Completed	Primary outcome was 6 month PFS. Participants with recurrent Grade 4 malignant glioma (ie, glioblastoma [GBM]) who were bevacizumab-naive; received bevacizumab - 11% PFS. Participants with recurrent Grade 4 malignant glioma (ie, glioblastoma [GBM]) who were bevacizumab-naive; received lenvatinib capsules - 21.2% PFS. Participants with recurrent Grade 3 malignant glioma who were bevacizumab-naive; received lenvatinib - 8% PFS. Participants with recurrent GBM who had disease progression following prior bevacizumab treatment; received lenvatinib - 7.6% PFS.
Bafetinib in	City of Hope Medical	NIH	Phase	Complet	

Treating Patients With Recurrent High-Grade Glioma or Brain Metastases	Center|National Cancer Institute (NCI)		1	ed	
Everolimus, Temozolomide, and Radiation Therapy in Treating Patients With Newly Diagnosed Glioblastoma Multiforme	Radiation Therapy Oncology Group|National Cancer Institute (NCI)|NRG Oncology	NIH	Phase 1|Phase 2	Completed	Combining everolimus with conventional chemoradiation leads to increased treatment-related toxicities and does not improve PFS in patients with newly diagnosed glioblastoma. Although the median survival time in patients receiving everolimus was comparable to contemporary studies, it was inferior to the control in this randomized study.
EGFR Inhibition Using Weekly Erlotinib for Recurrent Malignant Gliomas	Andrew B Lassman, MD|Genentech, Inc.|OSI Pharmaceuticals|Columbia University	Industry	Phase 1	Completed	
AZD8055 for Adults With Recurrent Gliomas	National Cancer Institute (NCI)|National Institutes of Health Clinical Center (CC)	NIH	Phase 1	Completed	
Phase I-II Everolimus and Sorafenib in Recurrent High-Grade Gliomas	National Cancer Institute (NCI)|National Institutes of Health Clinical Center (CC)	NIH	Phase 1|Phase 2	Completed	Median survival was 7.75 mo in BEV-naive GBM, 4.77 mo in BEV-treated GBM, and 11.97 mo in anaplastic astrocytoma
Lapatinib With Temozolomide and Regional Radiation Therapy for Patients With Newly-Diagnosed Glioblastoma Multiforme	Jonsson Comprehensive Cancer Center|GlaxoSmithKline|Novartis	Industry	Phase 2	Active, not recruiting	Pulse high-dose lapatinib in addition to standard therapy for newly-diagnosed GBM is a tolerable and safe regimen, but higher rates of lymphopenia should be noted.
Sorafenib, Valproic Acid, and Sildenafil in Treating Patients With Recurrent High-Grade Glioma	Virginia Commonwealth University|National Cancer Institute (NCI)	NIH	Phase 2	Active, not recruiting	Primary outcome number of patients with 6 month PFS was 8/47. 25/47 did not meet 6 month PFS. 14/47 were not evaluable.
Lapatinib Ditosylate Before Surgery in Treating Patients With Recurrent High-Grade Glioma	National Cancer Institute (NCI)	NIH	Phase 1	Active, not recruiting	
Study to Evaluate Safety and Activity of Crizotinib With Temozolomide and Radiotherapy in Newly Diagnosed Glioblastoma		Industry	Phase 1	Completed	

Perifosine and Torisel (Temsirolimus) for Recurrent/Progressive Malignant Gliomas	Andrew B Lassman, MD|Pfizer|AEterna Zentaris|Columbia University	Industry	Phase 1	Completed	
Study of LY2228820 With Radiotherapy Plus Concomitant TMZ in the Treatment of Newly Diagnosed Glioblastoma	Centre Jean Perrin|National Cancer Institute, France|ARC Foundation for Cancer Research	Other	Phase 1|Phase 2	Completed	
Study of Tesevatinib Monotherapy in Patients With Recurrent Glioblastoma	Kadmon Corporation, LLC	Industry	Phase 2	Completed	22.5% overall PFS6, with 25% in EGFR amplified and 18.2% in EGFRvIII gliomas
Dabrafenib and/or Trametinib Rollover Study	Novartis Pharmaceuticals|Novartis	Industry	Phase 4	Recruiting	
Ruxolitinib With Radiation and Temozolomide for Grade III Gliomas and Glioblastoma	Case Comprehensive Cancer Center	Other	Phase 1	Active, not recruiting	
A Trial of Ipatasertib in Combination With Atezolizumab	Institute of Cancer Research, United Kingdom|Hoffmann-La Roche	Industry	Phase 1|Phase 2	Recruiting	
18F-FDG PET and Osimertinib in Evaluating Glucose Utilization in Patients With EGFR Activated Recurrent Glioblastoma	Jonsson Comprehensive Cancer Center|AstraZeneca	Industry	Phase 2	Active, not recruiting	Treated patients exhibited a reduced glycolytic flux as indicated by reduction in 18F-FDG PET uptake (-3% change in SUV), tumor acidity (-19% change in MTRasym@3ppm) on pH-weighted CEST MRI, and glycolytic index (GI) (-25% change) in EGFR amplified recurrent GBM within 24 hours of treatment.
9-ING-41 in Patients With Advanced Cancers	Actuate Therapeutics Inc.	Industry	Phase 2	Recruiting	
Nedisertib and Radiation Therapy, Followed by Temozolomide for the Treatment of Patients With Newly Diagnosed MGMT Unmethylated Glioblastoma or Gliosarcoma	M.D. Anderson Cancer Center|National Cancer Institute (NCI)	NIH	Phase 1	Recruiting	

Tofacitinib in Recurrent GBM Patients	University of Texas Southwestern Medical Center|Pfizer	Industry	Phase 3	Recruiting	
DETERMINE Trial Treatment Arm 5: Vemurafenib in Combination With Cobimetinib in Adult Patients With BRAF Positive Cancers.	Cancer Research UK|University of Manchester|University of Birmingham|Royal Marsden NHS Foundation Trust|Hoffmann-La Roche	Industry	Phase 2|Phase 3	Recruiting	
Superselective Intra-arterial Cerebral Infusion of Temsirolimus in HGG	Nader Sanai|Barrow Neurological Institute|Ivy Brain Tumor Center|St. Joseph's Hospital and Medical Center, Phoenix	Other	Early Phase 1	Recruiting	
**Microenvironmental Targets (angiogenesis, cell-cell adhesion, iron/cation regulation)**					
Gefitinib Plus Temozolomide in Treating Patients With Malignant Primary Glioma	Sidney Kimmel Comprehensive Cancer Center at Johns Hopkins|National Cancer Institute (NCI)	NIH	Phase 1	Completed	For patients on anti-epileptic drugs, the MTD of gefitinib was 1,000 mg/day in combination with temozolomide. Dose-limiting toxicity (DLT) was due to diarrhea, nausea and vomiting. For patients not on anti-epileptic drugs, the MTD was 250 mg/day in combination with temozolomide. The DLT was due to increases in liver transaminases.
Safety and Efficacy Study of Tarceva, Temodar, and Radiation Therapy in Patients With Newly Diagnosed Brain Tumors	University of California, San Francisco|Genentech, Inc.	Industry	Phase 2	Completed	Median survival was 19mo, median PFS was 8.2 mo
Erlotinib and Temsirolimus in Treating Patients With Recurrent Malignant Glioma	National Cancer Institute (NCI)	NIH	Phase 1|Phase 2	Completed	1/12 patients treated with maximally tolerated dose with partial response at 8 weeks
Temozolomide and Radiation Therapy With or Without Vatalanib in Treating Patients With Newly Diagnosed Glioblastoma Multiforme	European Organisation for Research and Treatment of Cancer - EORTC	Other	Phase 1|Phase 2	Completed	
Imatinib Mesylate, Vatalanib, and Hydroxyurea in	Duke University|National Cancer Institute (NCI)	NIH	Phase 1	Completed	

Treating Patients With Recurrent or Relapsed Malignant Glioma					
Cetuximab, Bevacizumab and Irinotecan for Patients With Malignant Glioblastomas	Rigshospitalet, Denmark|Aalborg University Hospital|Odense University Hospital	Other	Phase 2	Completed	
PTK787/ZK 222584 in Combination With Temozolomide and Radiation in Patients With Glioblastoma Taking Enzyme-Inducing Anti-Epileptic Drugs	Massachusetts General Hospital|Dana-Farber Cancer Institute|Novartis	Industry	Phase 1	Completed	13/19 patients evaluable for a radiographic response, 2 had a partial response and 9 had stable disease. Vatalanib significantly increased PlGF and sVEGFR1 in plasma circulation and decreased sVEGFR2 and sTie2. Plasma collagen IV increased significantly by day 50 of treatment. Vatalanib was well tolerated.
Pazopanib In Combination With Lapatinib In Adult Patients With Relapsed Malignant Glioma	GlaxoSmithKline	Industry	Phase 2	Completed	Pazopanib and lapatinib can be given safely at single-drug doses
Phase (Ph) II Bevacizumab + Erlotinib for Patients (Pts) With Recurrent Malignant Glioma (MG)	Duke University|Genentech, Inc.	Industry	Phase 2	Completed	Bevacizumab plus erlotinib was adequately tolerated in recurrent MG patients. However, this regimen was associated with similar PFS benefit and radiographic response when compared with other historical bevacizumab-containing regimens.
Bevacizumab and Cediranib Maleate in Treating Patients With Metastatic or Unresectable Solid Tumor, Lymphoma, Intracranial Glioblastoma, Gliosarcoma or Anaplastic Astrocytoma	National Cancer Institute (NCI)	NIH	Phase 1	Completed	
Study of Bevacizumab Plus Temodar and Tarceva in Patients With Glioblastoma or Gliosarcoma	University of California, San Francisco	Other	Phase 2	Completed	Median overall survival was 19.8 months

Ph I Zactima + Imatinib Mesylate & Hydroxyurea for Pts w Recurrent Malignant Glioma	Annick Desjardins|Novartis Pharmaceuticals|AstraZeneca|Duke University	Industry	Phase 1	Completed	
Cediranib, Temozolomide, and Radiation Therapy in Treating Patients With Newly Diagnosed Glioblastoma	National Cancer Institute (NCI)	NIH	Phase 1|Phase 2	Completed	
Bevacizumab and Sorafenib in Treating Patients With Recurrent Glioblastoma Multiforme	Alliance for Clinical Trials in Oncology|National Cancer Institute (NCI)	NIH	Phase 2	Completed	Primary outcome 6 month PFS. 26.3% PFS in sorafenib 400 mg and 5 mg/kg bevacizumab group. 17.1% PFS in sorafenib 200 mg and 5mg/kg bevacizumab group.
RT, Temozolomide, and Bevacizumab Followed by Bevacizumab/Everolimus in First-line Treatment of GBM	SCRI Development Innovations, LLC|Genentech, Inc.|Novartis	Industry	Phase 2	Completed	This combination therapy regimen is safe and shows improved efficacy vs TMZ+RT
Afatinib (BIBW 2992) QTcF Trial in Patients With Relapsed or Refractory Solid Tumours	Boehringer Ingelheim	Industry	Phase 2	Completed	Objective response in 1/60 patients. PFS in 10.6/53 patients. Mean QTcF change -0.3 for 49 participants.
Bevacizumab and Erlotinib After Radiation Therapy and Temozolomide in Treating Patients With Newly Diagnosed Glioblastoma Multiforme or Gliosarcoma	Northwestern University|M.D. Anderson Cancer Center	Other	Phase 2	Completed	32/46 reached PFS12, and 4 achieved complete responses with average overall survival of 13.2 mo and 4 still alive at time of publication
Dasatinib and Bevacizumab in Treating Patients With Recurrent or Progressive High-Grade Glioma or Glioblastoma Multiforme	Alliance for Clinical Trials in Oncology|National Cancer Institute (NCI)	NIH	Phase 2	Completed	PFS6 was 29% in patients on BEV + dasatinib vs 18% for BEV alone
Temozolomide and Radiation Therapy With or Without Cediranib Maleate in Treating Patients With Newly Diagnosed	National Cancer Institute (NCI)|NRG Oncology|Radiation Therapy Oncology Group	NIH	Phase 2	Completed	Cediranib added to conventional therapy significantly increased the PFS6 rate in this GBM cohort

Glioblastoma					
Cediranib Maleate and Cilengitide in Treating Patients With Progressive or Recurrent Glioblastoma	National Cancer Institute (NCI)	NIH	Phase 1	Completed	
Gamma-Secretase Inhibitor RO4929097 and Cediranib Maleate in Treating Patients With Advanced Solid Tumors	National Cancer Institute (NCI)	NIH	Phase 1	Completed	
A Study of Avastin (Bevacizumab) and Irinotecan Versus Temozolomide Radiochemistry in Patients With Glioblastoma	Hoffmann-La Roche	Industry	Phase 2	Completed	79.31% bevacizumab and irinotecan with progression free survival at 6 months. 42.95% temozolomide with progression free survival at 6 months.
BIBF 1120 in Recurrent Glioblastoma Multiforme	Ulrik Lassen|Boehringer Ingelheim|University of Copenhagen|Rigshospitalet, Denmark	Industry	Phase 2	Completed	
BIBF 1120 for Recurrent High-Grade Gliomas	Patrick Y. Wen, MD|Boehringer Ingelheim|Wake Forest University Health Sciences|University of Virginia|Massachusetts General Hospital|The Cleveland Clinic|Dana-Farber Cancer Institute	Industry	Phase 2	Completed	No patients achieved PFS6
CAR T Cell Receptor Immunotherapy Targeting EGFRvIII for Patients With Malignant Gliomas Expressing EGFRvIII	National Cancer Institute (NCI)|National Institutes of Health Clinical Center (CC)	NIH	Phase 1|Phase 2	Completed	2/13 participants with treatment related adverse events (primary outcome).
Tivozanib for Recurrent Glioblastoma	Massachusetts General Hospital|National Comprehensive Cancer Network	Other	Phase 2	Completed	10% PFS6
A Randomized Phase II Clinical Trial on the Efficacy of Axitinib as a Monotherapy or in Combination With Lomustine for the Treatment of Patients With	Bart Neyns|Pfizer|Universitair Ziekenhuis Brussel	Industry	Phase 2	Completed	

Recurrent Glioblastoma					
Apatinib in Recurrent or Refractory Intracranial Central Nervous System Malignant Tumors	Rongjie Tao|Shandong Cancer Hospital and Institute	Other	Phase 2	Completed	
Safety Study of Afatinib for Brain Cancer	Santosh Kesari|Boehringer Ingelheim|Saint John's Cancer Institute	Industry	Phase 1	Completed	In total, 26 patients were screened and 24 were enrolled (median age 60, 78% male, 87.5% Caucasian, glioblastoma N=16, chordoma N=3, brain metastases N=2, meningioma N=1, anaplastic mixed oligoastrocytoma N=1). Patients had received a median of 2.5 prior treatments. Diarrhea (75%), rash (75%), nausea/vomiting (37.5%), fatigue (29.2%), anorexia (25%), and limb edema (16.7%) were the most common side effects. There were no CTCAE defined grade 4 toxicities. Grade 3 side effects infrequently occurred in the highest dosing cohort. These results demonstrate that pulsatile Afatinib at a dose of 280mg every 7 days is safe and tolerable for patients with brain involving cancers.
Clinical Trial on the Combination of Avelumab and Axitinib for the Treatment of Patients With Recurrent Glioblastoma	Universitair Ziekenhuis Brussel	Other	Phase 2	Completed	
Prediction of Therapeutic Response of Apatinib in Recurrent Gliomas	The First Affiliated Hospital of Zhengzhou University	Other		Recruiting	
Ketoconazole Before Surgery in Treating Patients With Recurrent Glioma or Breast Cancer Brain	Wake Forest University Health Sciences|National Cancer Institute (NCI)	NIH	Early Phase 1	Recruiting	

Metastases					
Anlotinib Combined With STUPP for MGMT Nonmethylated Glioblastoma	Second Affiliated Hospital, School of Medicine, Zhejiang University	Other	Phase 2	Recruiting	
**Cell Cycle/Apoptosis/Transcription Pathways**					
Study of the Poly (ADP-ribose) Polymerase-1 (PARP-1) Inhibitor BSI-201 in Patients With Newly Diagnosed Malignant Glioma	Sanofi	Industry	Phase 1|Phase 2	Completed	
Virus DNX2401 and Temozolomide in Recurrent Glioblastoma	Clinica Universidad de Navarra, Universidad de Navarra|DNAtrix, Inc.	Industry	Phase 1	Completed	
Trial of Ponatinib in Patients With Bevacizumab-Refractory Glioblastoma	Dana-Farber Cancer Institute	Other	Phase 2	Completed	No patients achieved PFS3
Safety and Efficacy of PD0332991 (Palbociclib), a Cyclin-dependent Kinase 4 and 6 Inhibitor, in Patients With Oligodendroglioma or Recurrent Oligoastrocytoma Anaplastic With the Activity of the Protein RB Preserved		Industry	Phase 2	Completed	
Zotiraciclib (TG02) Plus Dose-Dense or Metronomic Temozolomide Followed by Randomized Phase II Trial of Zotiraciclib (TG02) Plus Temozolomide Versus Temozolomide Alone in Adults With Recurrent Anaplastic Astrocytoma and Glioblastoma	National Cancer Institute (NCI)|National Institutes of Health Clinical Center (CC)	NIH	Phase 1|Phase 2	Completed	MTD was 250mg/d and PFS4 was 40%
Phase I/IIa Study of	Centre Francois	Other	Phase	Recruiti	

Concomitant Radiotherapy With Olaparib and Temozolomide in Unresectable High Grade Gliomas Patients	Baclesse|National Cancer Institute, France		1|Phase 2	ng	
A Phase 0 /II Study of Ribociclib (LEE011) in Combination With Everolimus in Preoperative Recurrent High-Grade Glioma Patients Scheduled for Resection	St. Joseph's Hospital and Medical Center, Phoenix|Ivy Brain Tumor Center|Barrow Neurological Institute	Other	Early Phase 1	Completed	Ribociclib exhibited good CNS penetration, and target modulation was indicated by inhibition of RB phosphorylation and tumor proliferation. Six of 12 patients were enrolled into the pharmacokinetic/pharmacodynamic-guided expansion cohort and demonstrated a median PFS of 9.7 weeks
BGB-290 and Temozolomide in Treating Isocitrate Dehydrogenase (IDH)1/2-Mutant Grade I-IV Gliomas	University of California, San Francisco|BeiGene USA, Inc.|Pacific Pediatric Neuro-Oncology Consortium	Industry	Phase 1	Recruiting	
Anticancer Therapeutic Vaccination Using Telomerase-derived Universal Cancer Peptides in Glioblastoma	Centre Hospitalier Universitaire de Besancon	Other	Phase 2	Recruiting	
B7-H3 CAR-T for Recurrent or Refractory Glioblastoma	Second Affiliated Hospital, School of Medicine, Zhejiang University|Ningbo Yinzhou People's Hospital|Huizhou Municipal Central Hospital|BoYuan RunSheng Pharma (Hangzhou) Co., Ltd.	Other	Phase 1|Phase 2	Recruiting	
**Immunotherapy Pathways**					
A Dose Escalation and Cohort Expansion Study of Anti-CD27 (Varlilumab) and Anti-PD-1 (Nivolumab) in Advanced Refractory Solid Tumors	Celldex Therapeutics|Bristol-Myers Squibb	Industry	Phase 1|Phase 2	Completed	GBM overall survival at 12 months was 40.9% Varlilumab and nivolumab were well tolerated, without significant toxicity beyond that expected for each agent alone. Clinical activity was observed in patients that are typically refractory to anti-PD-1 therapy, however, overall was not greater than expected for nivolumab monotherapy.
Ipilimumab and/or	National Cancer Institute	NIH	Phase	Complet	IPI and NIVO are safe and

Nivolumab in Combination With Temozolomide in Treating Patients With Newly Diagnosed Glioblastoma or Gliosarcoma	(NCI)|NRG Oncology		1	ed	tolerable with similar toxicity profiles noted with other cancers when given with adjuavant TMZ for newly diagnosed GBM.
Study of Cabiralizumab in Combination With Nivolumab in Patients With Selected Advanced Cancers	Five Prime Therapeutics, Inc.|Bristol-Myers Squibb	Industry	Phase 1	Complet ed	99.6% participants received 4 mg/kg cabiralizumab IV and 3 mg/kg nivolumab IV Q2W experienced adverse events. 52.1% participants in this group experienced severe adverse events.
Intra-tumoral Ipilimumab Plus Intravenous Nivolumab Following the Resection of Recurrent Glioblastoma	Universitair Ziekenhuis Brussel	Other	Phase 1	Recruiti ng	
Nivolumab for Recurrent or Progressive IDH Mutant Gliomas	Fabio Iwamoto, MD|Bristol-Myers Squibb|Columbia University	Industry	Phase 2	Active, not recruitin g	
Efficacy and Safety of Pembrolizumab (MK-3475) Plus Lenvatinib (E7080/MK-7902) in Previously Treated Participants With Select Solid Tumors (MK-7902-005/E7080-G000-224/LEAP-005)	Merck Sharp & Dohme LLC|Eisai Inc.	Industry	Phase 2	Active, not recruitin g	
Efficacy of Nivolumab for Recurrent IDH Mutated High-Grade Gliomas		Industry	Phase 2	Complet ed	At 24 weeks, 11/39 patients without disease free progression. Median PFS and OS were 1.84 (CI95% [1.81 ; 5.89]) and 14.7 months (CI95% [9.18; NR]), respectively. No patient definitively stopped Nivolumab due to side effects; the safety profile was consistent with prior studies of Nivolumab in gliomas and other cancers.
Trial of Anti-Tim-3 in Combination With Anti-PD-1 and SRS in Recurrent	Sidney Kimmel Comprehensive Cancer Center at Johns Hopkins|Novartis	Industry	Phase 1	Active, not recruitin g	

GBM	Pharmaceuticals				
Neoadjuvant Carilizumab and Apatinib for Recurrent High-Grade Glioma	Sun Yat-sen University	Other	Phase 2	Recruiting	
Ivosidenib (AG-120) With Nivolumab in IDH1 Mutant Tumors	Jason J. Luke, MD|Agios PharmaceuticalsInc.|Bristol-Myers Squibb|University of Pittsburgh	Industry	Phase 2	Recruiting	
**Other**					
A Phase 2b Clinical Study With a Combination Immunotherapy in Newly Diagnosed Patients With Glioblastoma	Imvax	Industry	Phase 2	Recruiting	IGV-001
